# Impact of solar-driven heating strategies on the phase change thermal storage performance of erythritol

**DOI:** 10.3389/fchem.2024.1330273

**Published:** 2024-01-23

**Authors:** Yuxuan Deng, Yu Zhen, Xiaojuan Zhu, Yanna Li, Jing Xu

**Affiliations:** ^1^ BaiLie School of Petroleum Engineering, Lanzhou City University, Lanzhou, China; ^2^ Tianxing Smart Control Technology Co., Ltd., Chengdu, China; ^3^ Chengdu Kaihang Technology Co., Ltd., Chengdu, China

**Keywords:** solar energy, erythritol, phase-change, heat storage, numerical calculation

## Abstract

With escalating energy demands, solar power stands out for its abundance and renewable advantages, presenting a paramount sustainable solution. Herein, we tactically incorporate phase change material (PCM) into solar energy systems, resulting in substantial enhancements in energy storage and utilization. Through numerical simulations, the thermal dynamics and phase change processes associated with various heating methodologies are investigated, aiming to achieve optimal thermal performance and energy efficiency. Detailed analysis of temperature dynamics within the PCM under two distinct heating methods reveals pivotal thermal fluctuations in both the PCM and water during heat release. The results indicate that bottom heating promptly induces rayleigh convection, resulting in a uniform temperature and a stable phase interface, which are desirable for heat transfer. In contrast, central tube heating concentrates heat transfer in the upper PCM layer, leading to an uneven phase interface and thermal stratification. Configurations with two horizontally aligned heating tubes result in a 36% reduction in melting duration compared to the single central tube setup, highlighting enhanced efficiency. Additionally, the bottom heating approach demonstrates improved energy storage efficiency in both the initial and second heating cycles. These findings highlight the potential of PCM-integrated combined heating systems for solar energy capture, confirming their efficiency and practicality in addressing modern household energy demands.

## 1 Introduction

The rising global energy demand has intensified concerns about a potential energy crisis (Semieniuk et al., 2021; Bhuiyan et al., 2022; Farghali et al., 2023). While conventional fossil fuels remain dominant, they pose significant challenges due to their finite availability and associated environmental consequences, particularly greenhouse gas emissions (Kalair et al., 2021; Arzaghi and Squalli, 2023; Wang and Azam, 2024). Solar energy, an environmentally friendly and sustainable option, is attracting increasing attention (Guney, 2016; Zou et al., 2023). The energy can be directly converted into electricity (Kruitwagen et al., 2021; El Hammoumi et al., 2022) or harnessed via photoelectrochemical processes to transform carbon dioxide into valuable chemicals, namely, methane and methanol (Alhuyi Nazari et al., 2021; Chen et al., 2021; Biswal et al., 2022). Advancements in solar technology have enhanced seawater desalination processes, thereby providing potable water to extensive populations (Memon et al., 2020; Wu et al., 2020; Wang et al., 2023). Moreover, developments in solar collectors and vacuum insulation technologies signify a transformative phase for sustainable infrastructure (Ren et al., 2021; Seddaoui et al., 2022). Thus, solar energy stands as a solution to the energy challenge and a symbol of environmental sustainability.

Photovoltaic energy conversion technology holds significant potential for various applications, including water purification, seawater desalination, high-temperature heterogeneous catalysis, antibacterial treatments, and de-icing (Xu et al., 2019; Zhou et al., 2019; Perović et al., 2020; Rotella Junior et al., 2021; Cheng et al., 2022). Despite considerable research, the practical implementation of this method remains a challenge. Castellani et al. assessed methane production via the Sabatier reaction and juxtaposed it with other CO_2_ valorization strategies, underscoring the potential of utilizing captured CO_2_ for solar energy storage (Castellani et al., 2017). Poupin et al. investigated the use of metal hydrides as thermoelectric materials for solar thermal energy storage (Poupin et al., 2020). Specifically, they utilized titanium manganese hydride for hydrogen storage and magnesium iron hydride as a high-temperature battery to ensure stable energy cycles. Kalidoss et al. optimized the energy storage performance of Therminol 55-TiO_2_ nanofluids by enhancing their solar energy conversion components (Kalidoss et al., 2020). Similarly, Yasmin et al. assessed the effectiveness of hybrid nanofluids for solar and thermal energy storage, highlighting their improved thermal and optical performance relative to conventional fluids (Yasmin et al., 2023). Phase change energy storage utilizes phase change materials (PCMs) to store and release energy during phase transitions, thereby enhancing energy efficiency for sustainable storage (Khan et al., 2016; Aftab et al., 2021; Hassan et al., 2022). Rao et al. designed a form-stable composite PCM combining magnesium nitrate hexahydrate with diatomite (Rao et al., 2018). Mahdi et al. evaluated paraffin wax as a PCM, focusing on its thermal storage capabilities (Mahdi et al., 2019). Nartowska et al. underscored the potential of sodium dihydrogen phosphate dodecahydrate as an eco-friendly and cost-effective PCM for solar devices (Nartowska et al., 2023). Wang et al. demonstrated that pairing foam aluminum with paraffin improves the PCM’s thermal conductivity (Wang et al., 2015). Meanwhile, Cui et al. posited that incorporating metal foam within PCMs enhances heat transfer rates (Cui et al., 2022). The unique capabilities of PCMs in absorbing and releasing thermal energy have rendered them instrumental, particularly in harnessing renewable energy sources such as solar power. Despite substantial advancements, the performance of PCMs in practical applications continues to be constrained by their thermal transfer efficiency and energy dispersal properties. In particular, varying heating strategies exert significant influences on the thermal conductivity and energy dispersion performance of PCMs. As such, elucidating the optimal heating methodology for PCMs remains a paramount area of inquiry in phase change thermal energy storage research.

In this study, we conceptualize the solar energy captured by the collector through a uniform heat flux density model. Additionally, we conduct a thorough investigation of a PCM with superior performance characteristics, focusing on evaluating its thermal storage efficiency across bottom and central tube strategies. The Solidification/Melting model effectively simulates phase transition phenomena, whereas the Volume of Fluid (VOF) model accurately delineates the interface between solid and liquid states in PCM. Our primary focus is on analyzing the phase transition, temperature fluctuations, and flow dynamics of the storage unit to ascertain the optimal thermal conversion efficiency. This comprehensive analysis extends to both the energy storage and release phases of the PCM, ensuring precise determination of the system’s optimal thermal conversion efficiency. The study furnishes a comprehensive reference framework that is pivotal for the development and integration of PCMs in solar energy applications.

## 2 Materials and methods

### 2.1 Physical model

**FIGURE 1:**
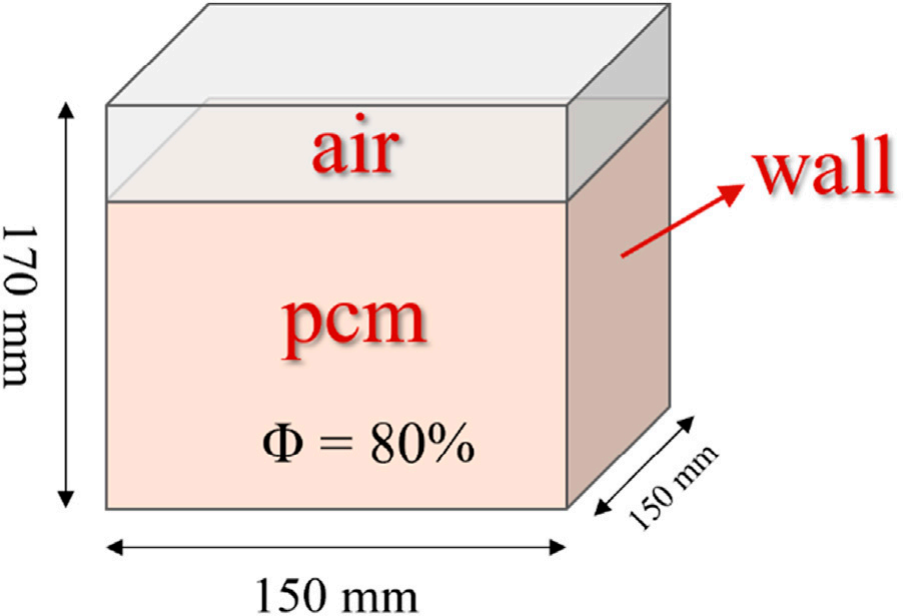
Cubic thermal storage model with 80% phase change material and distinct air layer above.

We employed a cubic thermal storage system surrounded by an external boundary, with an inner chamber measuring 150 mm × 150 mm × 170 mm, as illustrated in Figure 1. Within this chamber, a blend of PCM and air was introduced. Our previous research indicated that an 80% PCM concentration resulted in optimal energy storage efficiency (Deng et al., 2022). The system efficiently captures solar energy, directing it towards the bottom and central conduit, which consequently facilitates the PCM transition from solid to liquid, enhancing solar energy storage.

**FIGURE 2:**
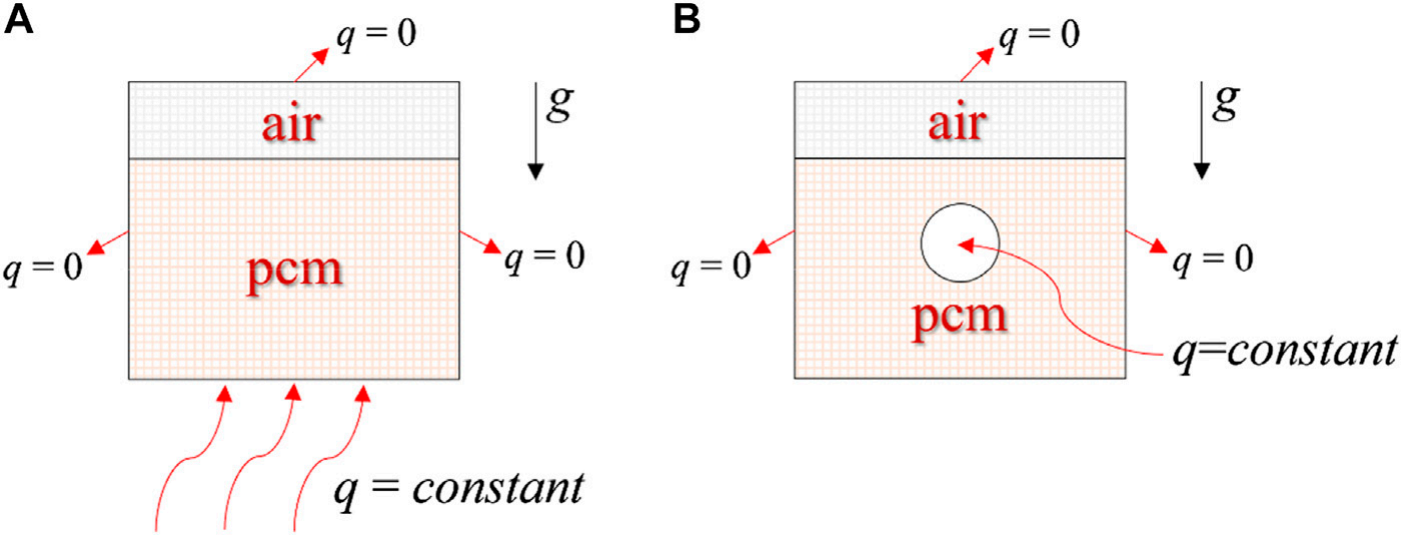
Simplified two-dimensional computational models. **(A)** Bottom heating; **(B)** Central tube heating.

Given the cube’s inherent symmetry, the model can be simplified. Figure 2 depicts a two-dimensional representation with dimensions of 150 mm by 170 mm. The bottom and central tube consistently demonstrated a heat flow density (q ≠ 0), while the remaining sides were adiabatic (q = 0).

### 2.2 Governing equation

The equation for continuity can be expressed as (Li et al., 2023):

$$\frac{\partial \rho }{\partial t}+\nabla \cdot u=0$$ (1)

where *ρ* is the density of PCM kg/m^3^, *u* is the PCM flow rate (m/s), and *t* is the time (s).

The equation for momentum was given as (Li et al., 2021):

$$\frac{\partial u}{\partial t}+\left(u\cdot \nabla \right)\left(u\right)=-\frac{1}{\rho }\nabla \left(P\right)+\frac{\mu }{\rho }\nabla^{2}u+\frac{1}{\rho }S$$ (2)

$$\left\{\begin{array}{c} S_{x}=\frac{{\left(1-\beta \right)}^{2}}{\left(\beta^{3}+\omega \right)}A_{mush}u\\ S_{y}=\frac{{\left(1-\beta \right)}^{2}}{\left(\beta^{3}+\omega \right)}A_{mush}u-\rho g\alpha \left(T-T_{ref}\right) \end{array}\right.$$ (3)

where *β* signifies the rate of the liquid phase, which lies between 0 and 1. *μ* stands for the dynamic viscosity and is expressed in Pa·S. The constant related to the paste-phase domain is represented as Amush, typically considered around 10^5^. To prevent the incorporation of a zero value in our equation, ω is utilized, with its common values ranging from 104 to 107. Lastly, α denotes the thermal expansion coefficient.

The equation for energy was given as (Yang et al., 2021):

$$\frac{\partial }{\partial x}\left(\rho H\right)+\nabla \left(\rho uH\right)=\nabla \cdot \left(k\nabla T\right)+S$$ (4)

$$\left\{\begin{array}{c} H=h+\Delta H\\ h=h_{ref}+\int_{T_{ref}}^{T}c_{p}dT\\ \Delta H=\beta L \end{array}\right.$$ (5)

The total enthalpy (H) per unit mass is denoted in kJ/kg, and it includes contributions from both the sensible enthalpy (h) and the latent heat (L). Meanwhile, *h*_ref_ represents the baseline enthalpy value. Furthermore, the PCM’s thermal behavior is characterized by its thermal conductivity (k), expressed in W/(m·K), and its constant pressure specific heat capacity (cp), with units J/(kg·K). Additionally, S stands for a pertinent source term in this formula.

### 2.3 Materials

The type of PCM and its thermal retention properties play a crucial role in determining the energy storage system’s heat retention density and thermal transfer efficiency. Table 1 lists standard medium-to-low-temperature PCMs and their physical properties (Fallahi et al., 2017; 31; Irfan Lone and Jilte, 2021; Yang et al., 2021; Sinaga et al., 2023). An examination of Table 1 reveals that erythritol has a latent heat value for phase transition of 339 kJ/kg. Consequently, using erythritol in phase-change thermal storage can reduce both the weight of the retention material and the storage system’s capacity while maintaining the same heat flux density. This leads to lower transportation costs per storage unit and enhances the economic viability of solar power systems. Given these benefits, this study focuses on erythritol.

**TABLE 1:** Physical parameters.

Number	Material	Phase-change temperature (°C)	Latent heat of phase change (kJ/kg)	Thermal conductivity (W/m·K)	Density (kg/m^3^)
1	Fatty acid	70.0	186.5	0.172	848
2	Barium hydroxide octahydrate	78.0	265.7	0.653	1,660
3	Naphthalene	80.0	147.7	0.132	976
4	Magnesium chloride hexahydrate	117	168.0	0.570	1,450
5	Erythritol	118	339.0	0.326	1,330

### 2.4 Numerical simulation

In our study, to enhance computational efficiency, we made the following assumptions: (1) An insulating layer surrounded the heat storage unit, significantly reducing heat loss to the surrounding environment; (2) A steady thermal flow rate was assumed for boundary conditions due to the continuous influence of the heat source on the storage unit’s base; (3) The PCM was considered homogeneous, and the trapped air was modeled as an ideal gas; (4) When erythritol transitioned to its liquid state, it behaved as an incompressible Newtonian fluid, and its buoyancy effects conformed to the Boussinesq approximation.

#### 2.4.1 Computational domain mesh

**FIGURE 3:**
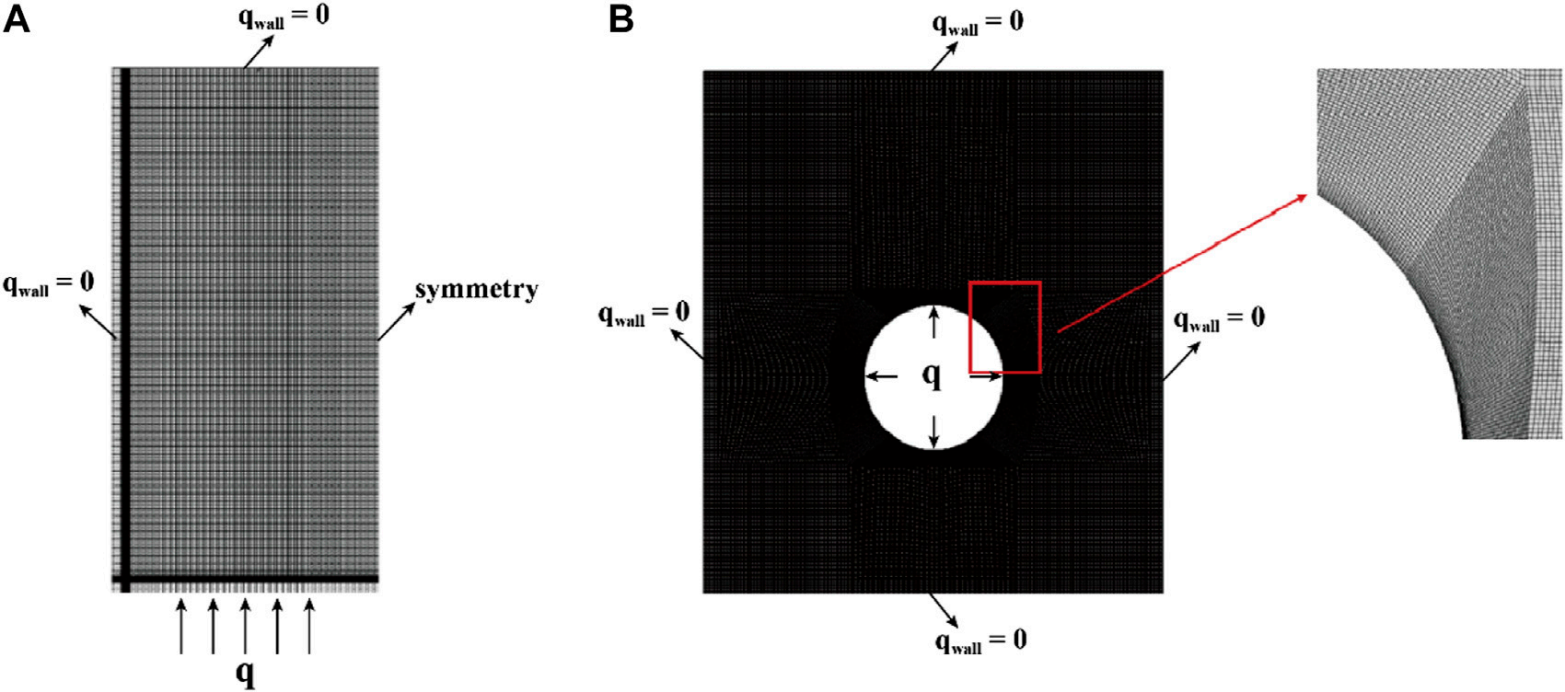
Simplifying two-dimensional computational models with discrete grids. **(A)** Bottom heating; **(B)** Central tube heating.

A structured mesh was generated for the two-dimensional computational domain using ICEM CFD software, as illustrated in Figure 3. The mesh layout for the bottom-heating configuration is symmetric, resembling half of a rectangle (Figure 3A). Notably, the bottom edge features finer grid lines, underscoring the emphasis on simulating heat accumulation in these regions. In contrast, the central-tube heating configuration showcases an entire rectangular grid (Figure 3B). This design is characterized by a denser grid at its center, highlighting the significance of heat flow and temperature distribution in that region. The increased granularity in the center underscores the precision required for modeling heat from a central source. The structured meshing approach accurately captures the various heating methods within the computational domain.

**FIGURE 4:**
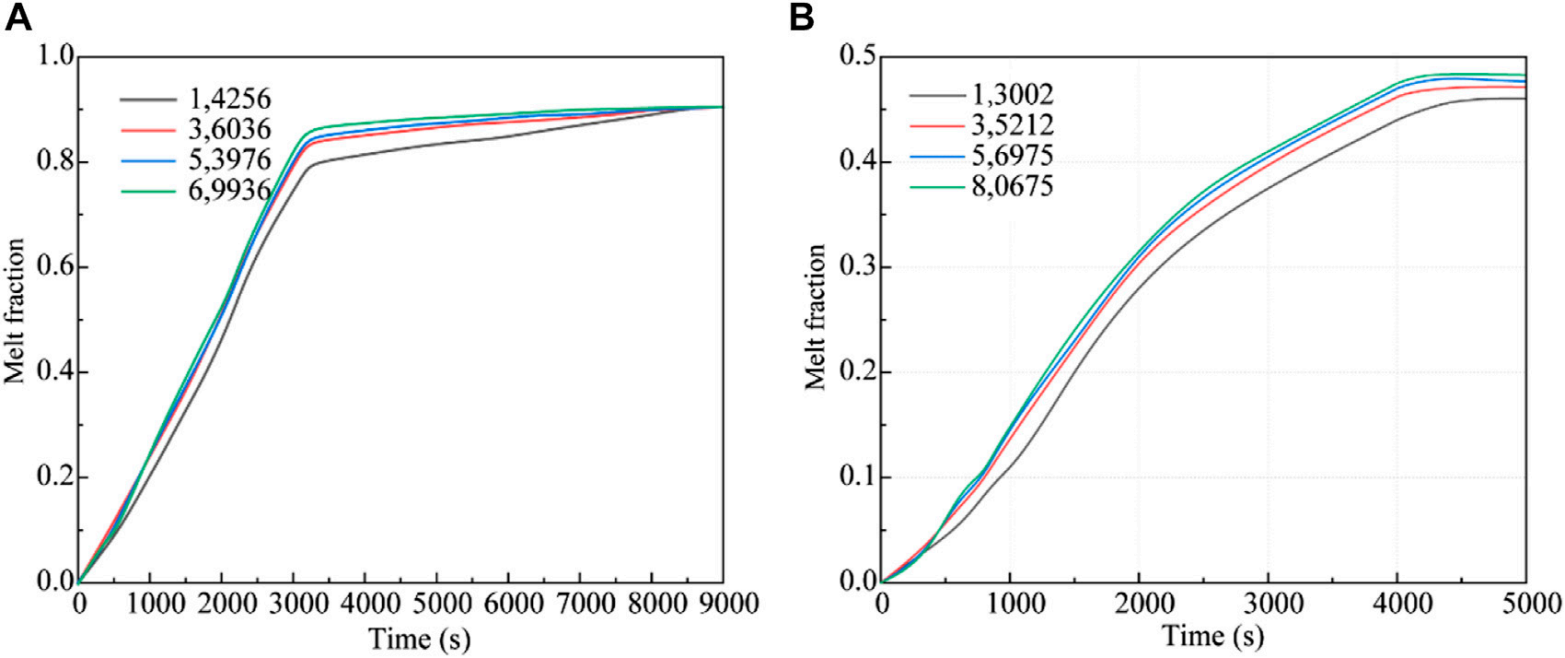
Grid-independence verification. **(A)** Bottom heating; **(B)** Central tube heating.

The impact of grid size was rigorously assessed in both the bottom heating and central tube heating methodologies. For the bottom heating method, an in-depth analysis ensured that the results were independent of grid size. The melting patterns of erythritol were studied across four grid configurations: 14,256, 36,036, 53,976, and 69,936. Transitioning from a grid count of 36,036 to either 53,976 or 69,936 led to variations in the liquid-phase volume fraction of erythritol between 5.72% and 6.72%, as illustrated in Figure 4A. These variations suggest a negligible impact of grid size on the results. Similarly, for the central tube heating method, the computational domain comprised four unique node sets with grid counts of 13,002, 35,212, 56,975, and 80,675. Simulations of these configurations, showcasing the variance in the PCM liquid-phase volume fraction over a 5000s duration, are displayed in Figure 4B. The results from the 13,002 grid configuration stood out as notably different from the others. To balance computational accuracy with resource constraints, we chose the 36,036 grid for the bottom heating analysis and the 35,212 grid for the central tube heating simulations.

#### 2.4.2 Boundary conditions and setting

To accurately model the phase-change behavior of erythritol in our thermal storage system, we utilized a two-dimensional, transient, and implicit solver, incorporating the Solidification/Melting and VOF functionalities in ANSYS Fluent. The momentum and energy equations were discretized using a second-order upwind scheme. For pressure gradient discretization, we chose the PRESTO scheme. The SIMPLE algorithm facilitated pressure-velocity coupling. To enhance convergence, we adjusted the partial relaxation factor to 0.5 and set a time increment of 0.01 s. A type-two boundary condition ensured a constant heat flux (q = 20,000 W/m^2^), while the other surfaces maintained adiabatic (q = 0). The system began with a uniform temperature of T_0_ = 298 K. The phase-change properties of erythritol are detailed in Table 1.

### 2.5 Experiment test

**FIGURE 5:**
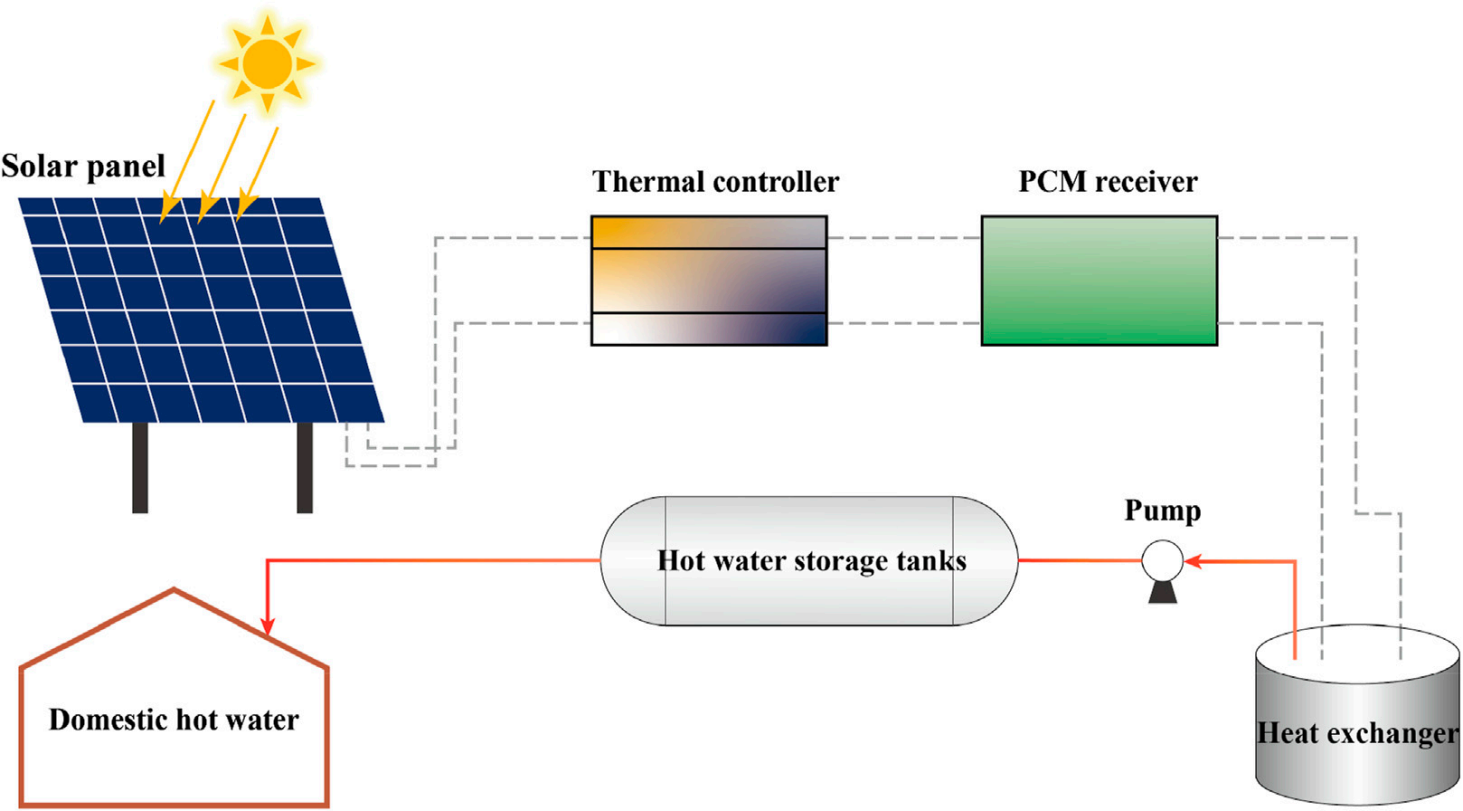
Schematic of solar thermal system: details a solar panel linked to a PCM receiver via a thermal controller, connected to hot water storage and a heat exchanger for domestic use.

To evaluate the efficacy of solar energy storage with PCM in water heating systems, a practical test was conducted. Solar panels were utilized under optimal illumination to capture thermal energy. The energy was subsequently regulated by a thermal controller to ensure its proper conveyance to the PCM receiver, where the material transitioned phases to store solar energy effectively. Simulating typical domestic hot water usage, a pump conveyed the accumulated heat to a heat exchanger, subsequently releasing it to elevate the water temperature in the storage tanks. The hot water was subsequently drawn from the tanks, replicating standard domestic uses such as bathing and washing. The temperature of the PCM was monitored during storage and release of heat, as well as the temperature of the water during the release of heat. The energy storage process is shown in Figure 5. This test aimed to ascertain the efficiency and reliability of the system setup and to identify potential optimization pathways for enhanced performance.

## 3 Results and discussion

### 3.1 Model validation

**FIGURE 6:**
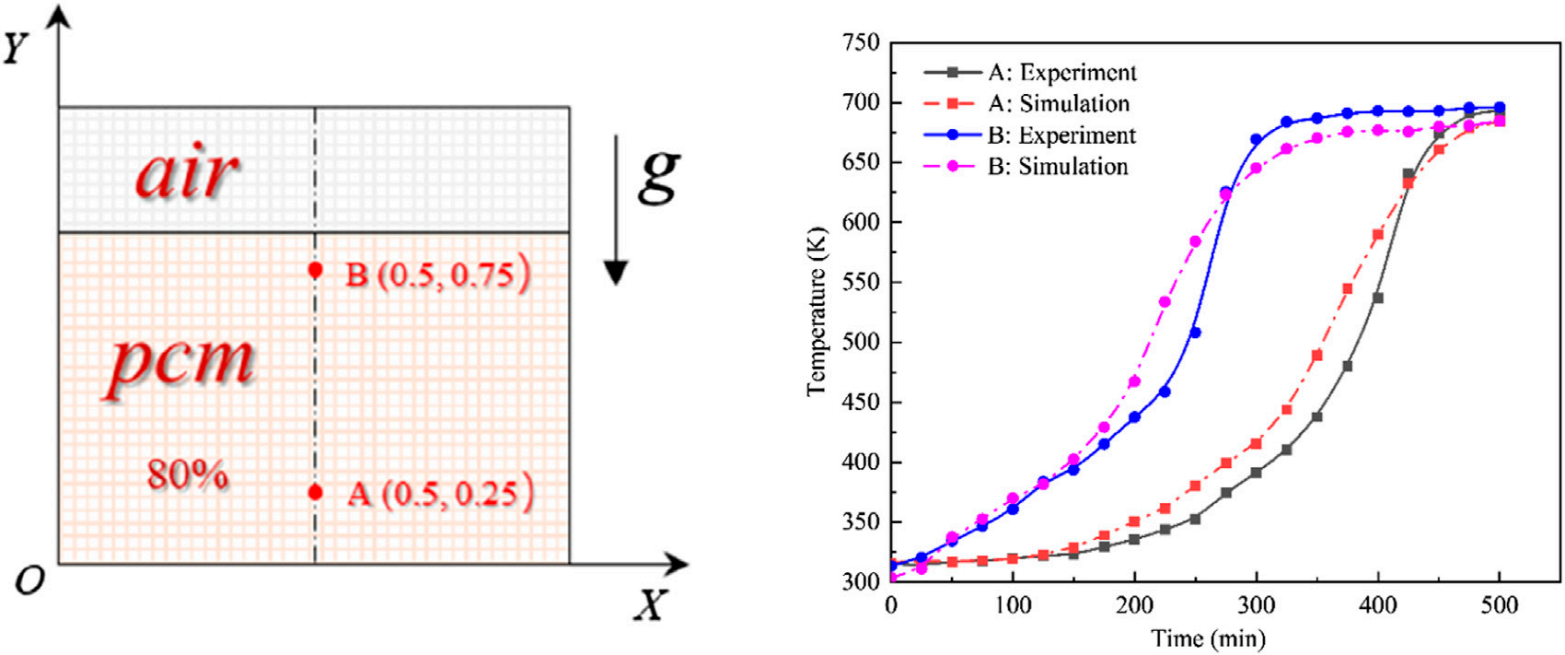
Comparison of experimental vs. simulated results.

In evaluating the reliability of computational results, we compared temperatures at two specific points, A (0.5, 0.25) and B (0.5, 0.75), located on the apparatus’s central axis, with numerical simulation results (Figure 6). Initial comparisons showed a close alignment between experimental and simulated temperatures at the beginning of melting. As the heating continued, each layer of PCM successively reached its melting point, starting from the bottom and ending at the top. Consistent temperatures were observed across all layers when the PCM was fully melted, indicating a synchronized increase. However, differences between the simulated and experimental values became evident during the mid to late heating stages. These differences were ascribed to natural convective flows and superheating events noted in our experiments. Still, the general temperature trends aligned closely. Subsequently, the discrepancies at points A and B measured 1.6% and 1.8%, respectively. Given these slight deviations, which fall within acceptable limits, we confirm the validity of our results.

### 3.2 Influence of the bottom heating method on the thermal-storage process

**FIGURE 7:**
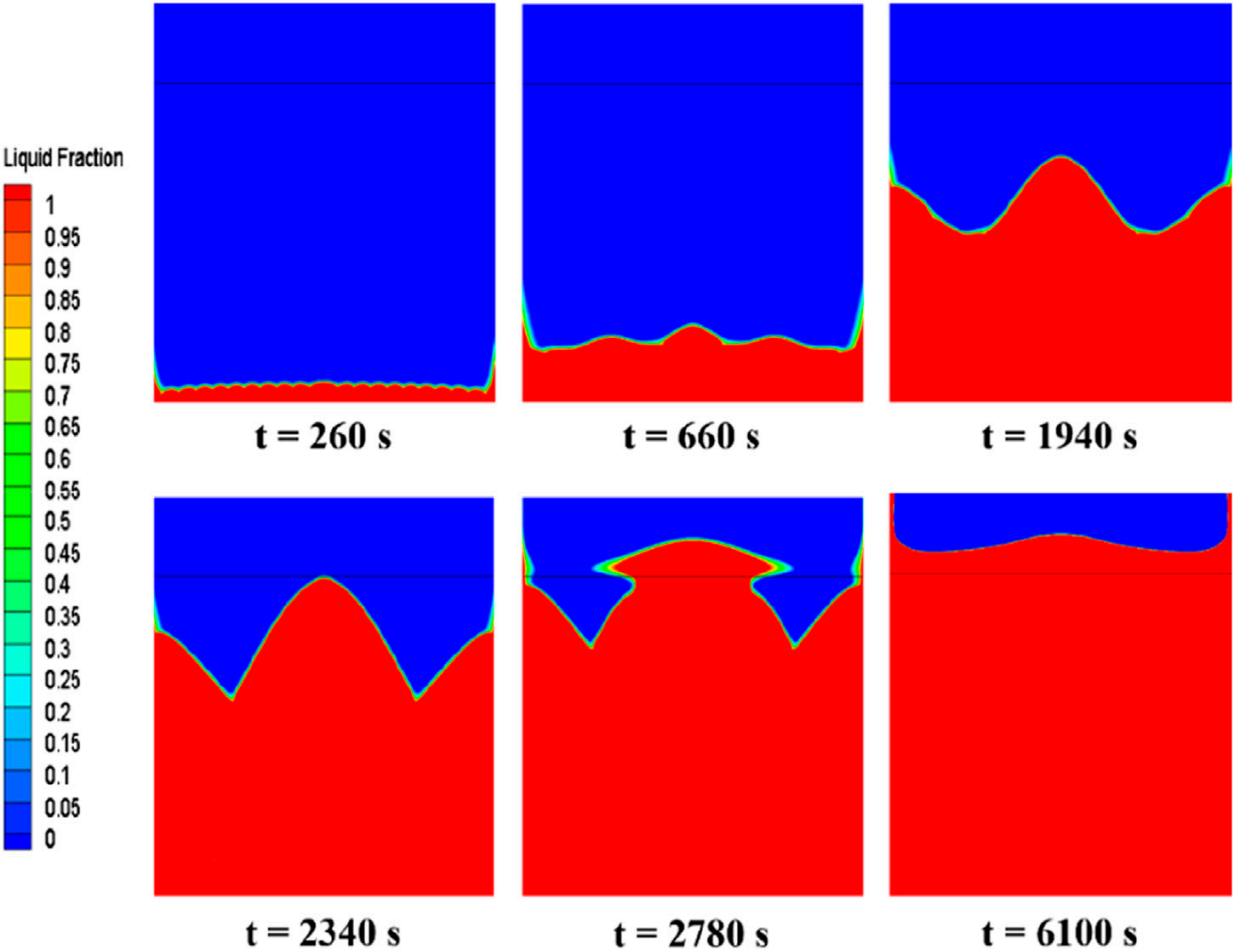
Phase-interface distribution of erythritol at different moments during bottom heating.

Figure 7 depicts the evolution of the erythritol phase interface within a rectangular cavity energy storage device at various melting intervals. Initially, the PCM near the bottom begins to melt, with the melting process propagating upwards from the bottom faster along the sidewalls than in the center. Over time, the liquid PCM area expands, enhancing natural convection and altering the solid-liquid phase interface. At t = 260 s, a thin liquid region appears at the device’s base, displaying small, regular wave patterns on the solid-liquid phase interface due to Rayleigh-Bénard convection. Its density decreases as the newly melted PCM at the bottom heats up. Consequently, this lighter PCM rises, forming regular convective or Bénard cells, accounting for the observed ripples. As the liquid phase area amplifies the convection effect, the Bénard cells coalesce into larger vortices, concurrently diminishing the frequency of interface ripples while augmenting their amplitude (t = 660 s). Subsequently, the interface assumes a W-shape, which appears smoother and denotes intensified natural convection (t = 1940 s and t = 2,340 s). As time progresses, most of the solid PCM undergoes melting, and the liquid phase proliferates beyond the initial air layer-PCM interface, engendering a “mushroom” shape that progressively melts the residual solid PCM (t = 2,780 s). Ultimately, the PCM becomes entirely molten, exhibiting a nearly horizontal phase interface, a manifestation of consistent heating and marking the cessation of the latent heat storage process (t = 6,100 s).

**FIGURE 8:**
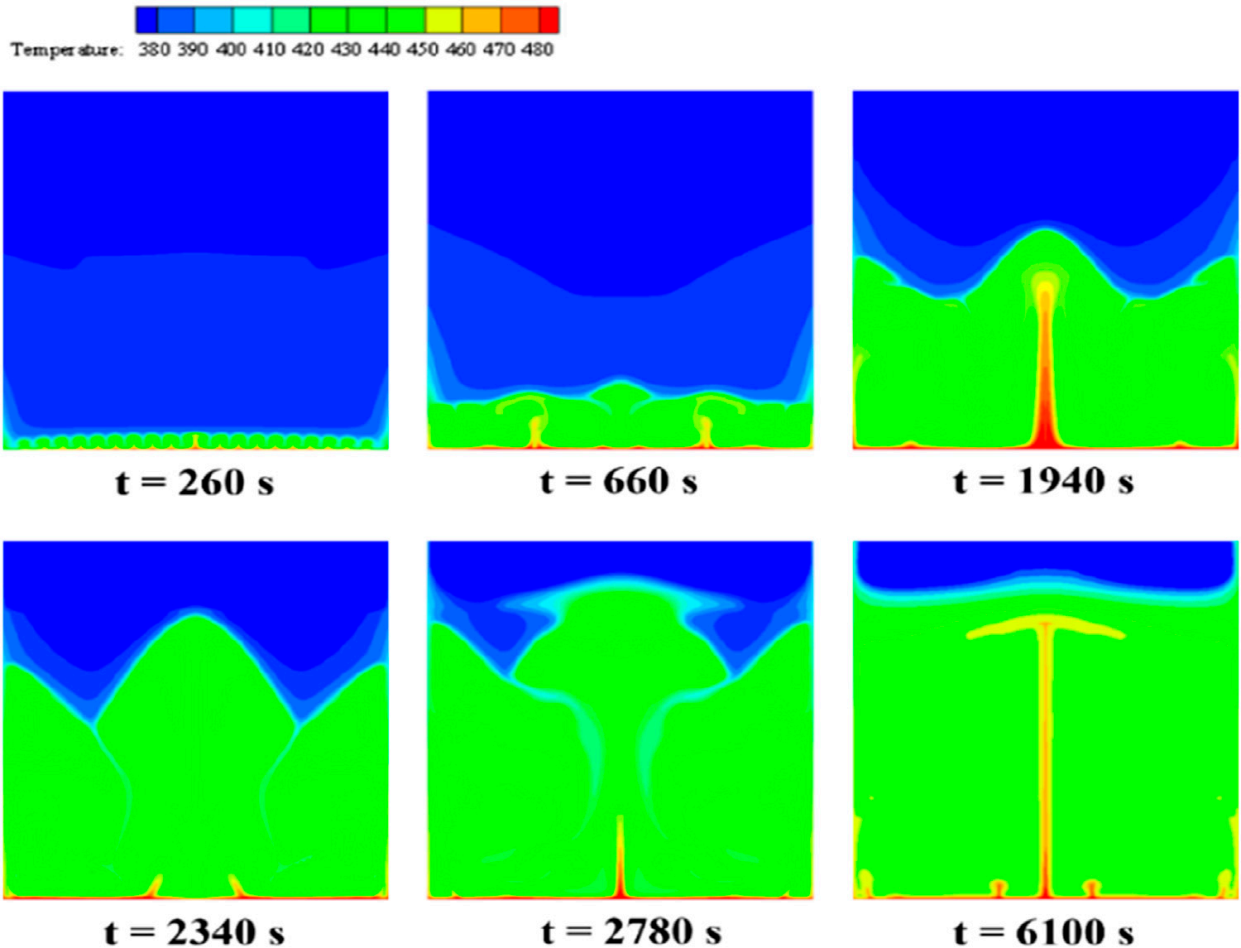
Temperature distribution of erythritol at different moments during bottom heating.

Figure 8 illustrates the temperature contours of erythritol during its melting intervals, aligning with the PCM phase interface distribution from Figure 7. The temperature of the PCM closest to the bottom heating wall rises initially and remains higher than that of the PCM situated further away. At the same horizontal level, the temperature near the sidewalls is higher than in the central regions. By t = 260 s, the temperature of the thin layer of PCM adjacent to the bottom heating element reaches approximately 420 K, surpassing its melting threshold, thereby converting this erythritol segment from solid to liquid. Heat ascends through the liquid PCM, creating a linear temperature gradient as the bottom PCM heats up. This process transforms the initially erratic heat movement into a macroscopic structured pattern, manifesting as distinct convection cells, which produce a consistently undulating temperature distribution. More PCM within the device exceeds its melting point, enhancing temperature uniformity near the bottom and forming a “jet-like” pattern in two regions (t = 660 s). A columnar high-temperature zone along the central axis forms a W-shaped profile at the phase interface, while the adjacent thermal layer presents a diminished gradient (t = 1940 s). The W-shaped profile persists, with colder zones in its concavities due to increased liquid-phase PCM, while the motion of the PCM, driven by buoyancy and gravity, creates two cold zones (t = 2,340 s). Finally, a consistent horizontal temperature distribution appears when the device contains no solid PCM. With heat flux density heating, the area closest to the bottom heating element continues to increase temperature, ultimately reaching a stabilized state with enhanced heat exchange uniformity in the liquid PCM (t = 6,100 s).

**FIGURE 9:**
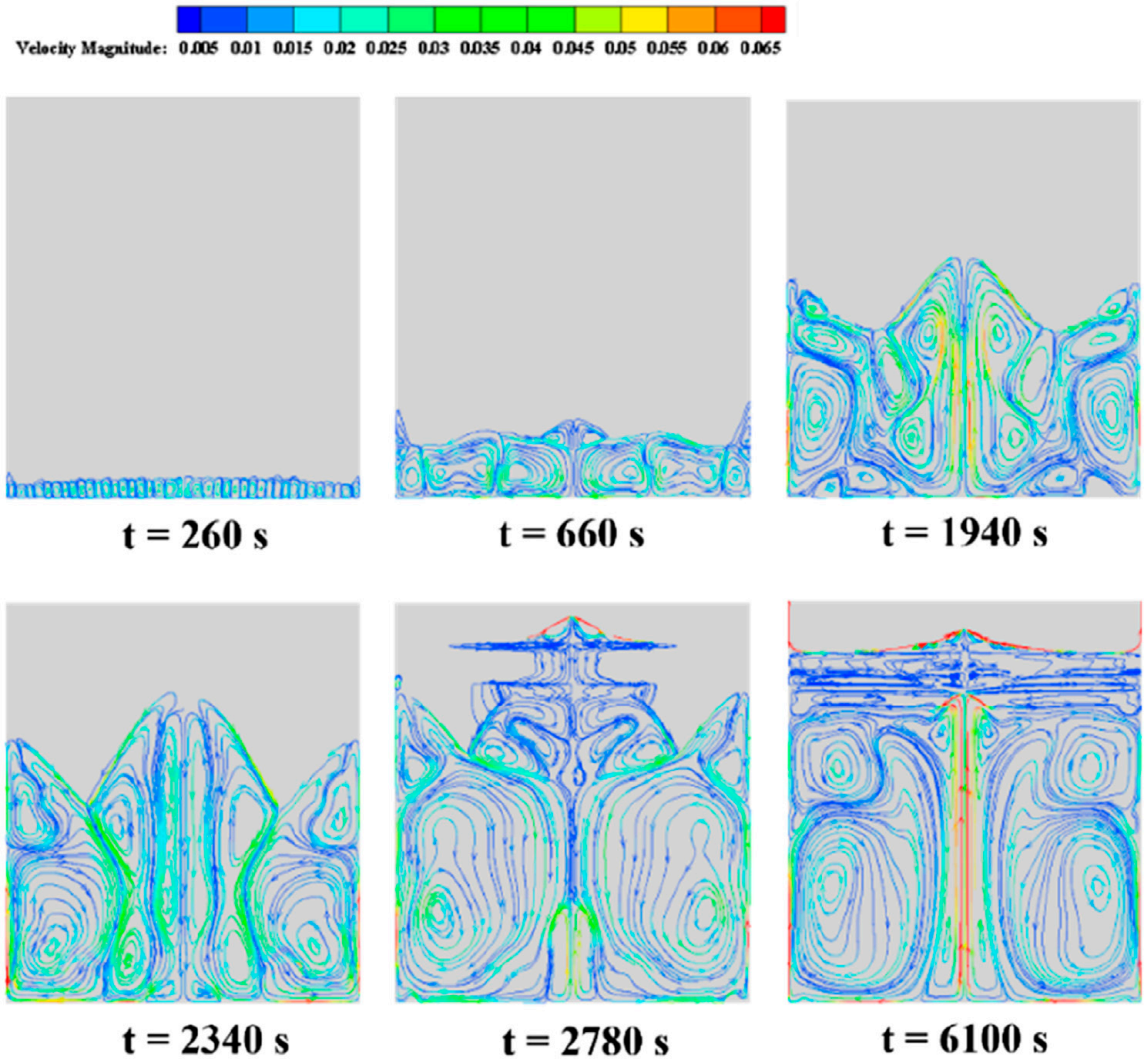
Velocity streamline of erythritol at different moments during bottom heating.

Figure 9 illustrates the evolution of velocity streamline patterns during the erythritol melting process in a rectangular cavity energy storage device, with the color spectrum representing velocity variations from blue (low) to red (high). As the PCM melts, the vortex dynamics exhibit a notable transformation in both count and size, influenced by the Rayleigh convection phenomenon due to bottom heating and resultant convection cells. At t = 260 s, a proliferation of diminutive vortices is apparent at the device’s base. These vortices exhibit peak velocities at their centers, diminishing toward their peripheries. This pattern arises from the elevated fluid temperature due to bottom heating, engendering consistent convection cells attributed to the Rayleigh convection phenomenon. This convection induces a rotation in these cells, producing alternating vortices. By t = 660 s, the base vortices grow in magnitude while their number reduces. The interface between the heating wall and a vortex exhibits an augmented velocity and temperature. Notably, the boundary of the solid-liquid transition aligns with the streamline or natural convection boundary, suggesting the shaping influence of natural convection on the phase interface. Twin vortices with opposing rotations appear (t = 1940 s), followed by dissipation of the central high-temperature zone (t = 2,340 s). More giant, counter-rotational vortices develop as the liquid phase enlarges (t = 2,780 s), with twin symmetrical vortices emerging centrally at the base (t = 6,100 s). The interface of these vortices indicates peak velocity and temperature.

### 3.3 Influence of the central tube heating method on the thermal-storage process

**FIGURE 10:**
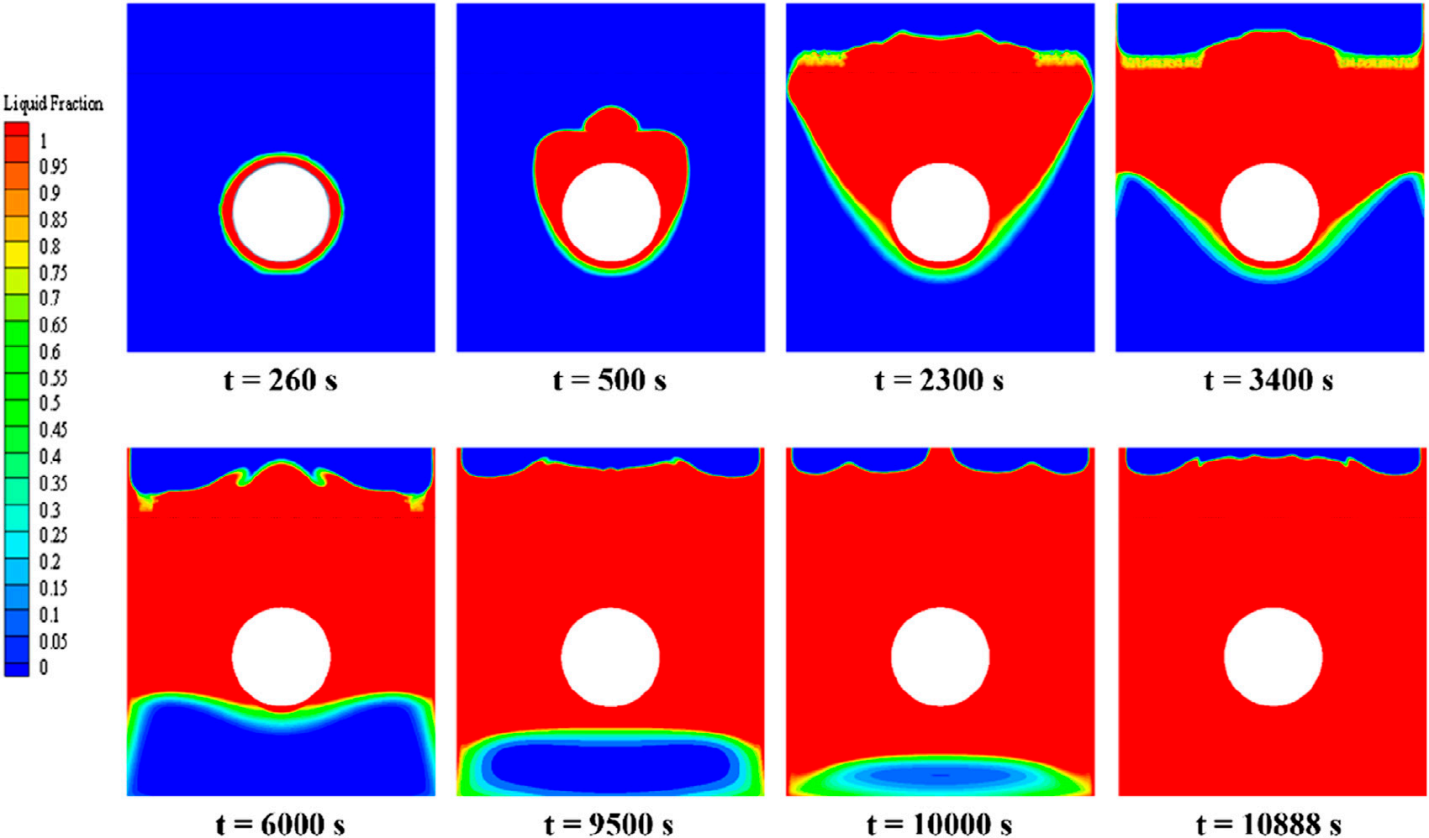
Phase-interface distribution of erythritol at different moments during central tube heating.

Figure 10 illustrates the distribution of phase interfaces in the PCM during its melting stages under central tube heating. Initially, the PCM closest to the tube transforms from solid to liquid due to direct heat transfer, beginning the phase change symmetrically. However, an asymmetrical distribution emerges as natural convection causes the lighter liquid PCM to rise (t = 500 s). As melting progresses, a distinct liquid phase region forms around the heating tube since areas closer to the heating source reach higher temperatures, thus accelerating their melting rate compared to more distant areas. The liquid phase, influenced by continuous heat supply and convection, expands horizontally and vertically, surpassing its initial boundary (t = 2,300 s). Two clear phase interfaces emerge, indicating varying melting rates within the PCM due to temperature disparities (t = 3,400 s). Continuous heating further melts the solid PCM, primarily near the tube (t = 6,000 s). The phase interface at the base takes on an elliptical shape, influenced by the liquid PCM’s buoyant flow against the solid PCM’s resistance, leading to a mixed mushy zone (t = 9,500 s). Finally, the entire PCM is melted, underscoring the efficiency of the central tube heating and the achievement of thermal equilibrium (t = 10,888 s).

**FIGURE 11:**
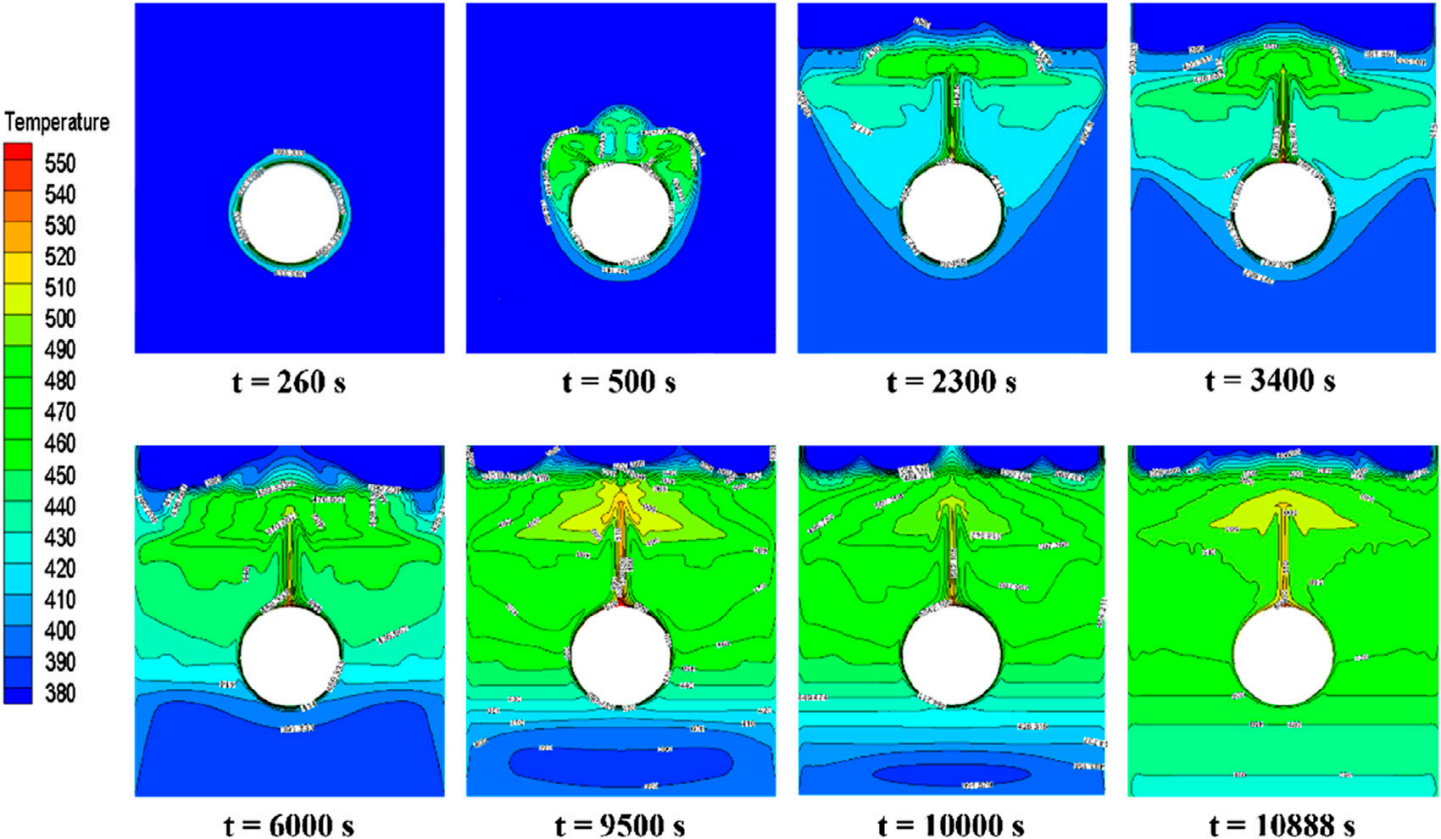
Temperature distribution of erythritol at different moments during central tube heating.

Figure 11 depicts the temperature contours of the PCM during central tube heating. Initially, a distinct temperature gradient radiates from the central line, suggesting conduction as the primary heat transfer mode (t = 240 s). As the phase transition advances, unevenly extending isotherms towards the upper central tube indicate the emergence of natural convection, driven by temperature disparities and the resulting fluid motion (t = 500 s). A narrow band of elevated temperature contours persists on the central heating tube’s surface. This stratified temperature profile might be attributed to the buoyant rise of the warmer liquid PCM and the concurrent sinking of the cooler PCM, resulting in a layered thermal distribution (t = 2,300 s). Subsequently, the liquid PCM then moves laterally towards the device sidewalls and descends, possibly driven by the interplay between the buoyancy of the liquid PCM and the resistance from the solid phase, broadening the isothermal region in the liquid phase (t = 3,400 s). Below the central tube, isotherms predominantly align horizontally, implying a reduced influence of natural convection on the melting PCM below (t > 6,000 s). This behavior might arise from alterations in the density and flow characteristics of the PCM in that region. As melting advances, the temperature differential within the liquid PCM diminishes, manifesting a more uniform temperature distribution, indicative of enhanced heating efficacy and balanced heat propagation.

**FIGURE 12:**
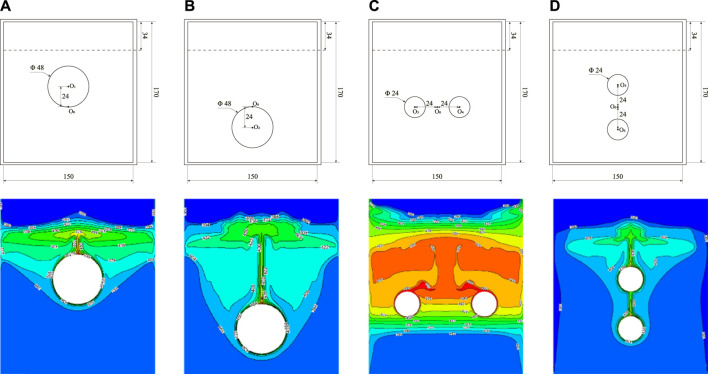
Temperature distribution under different heating tube arrangements. **(A)** Center point shifted upward 24 mm, **(B)** Center point shifted downward 24 mm, **(C)** Horizontal arrangement of double tubes, **(D)** Vertical arrangement of double tubes.

Figure 12 displays the temperature distribution at 3,000 s during the melting process under different heating tube configurations. The illustration emphasizes that the PCM directly above any heating tube undergoes the most rapid temperature rise. When a single heating tube is used, its close position to the bottom produces a broader region of heightened PCM temperature within the storage unit, leading to an expansive isothermal zone within the liquid PCM. In configurations with dual heating tubes, the horizontal arrangement facilitates more rapid heat distribution throughout the PCM than the vertical arrangement. This results in the PCM at the device’s top almost entirely transitioning to its liquid state, indicated by temperatures exceeding its melting point and a consistent temperature profile at the base. In contrast, devices with vertically aligned heating tubes demonstrate less efficient heat spread, manifesting a narrower high-temperature zone at the top. Optimal heat transfer is observed with a pair of horizontally aligned heating tubes.

**FIGURE 13:**
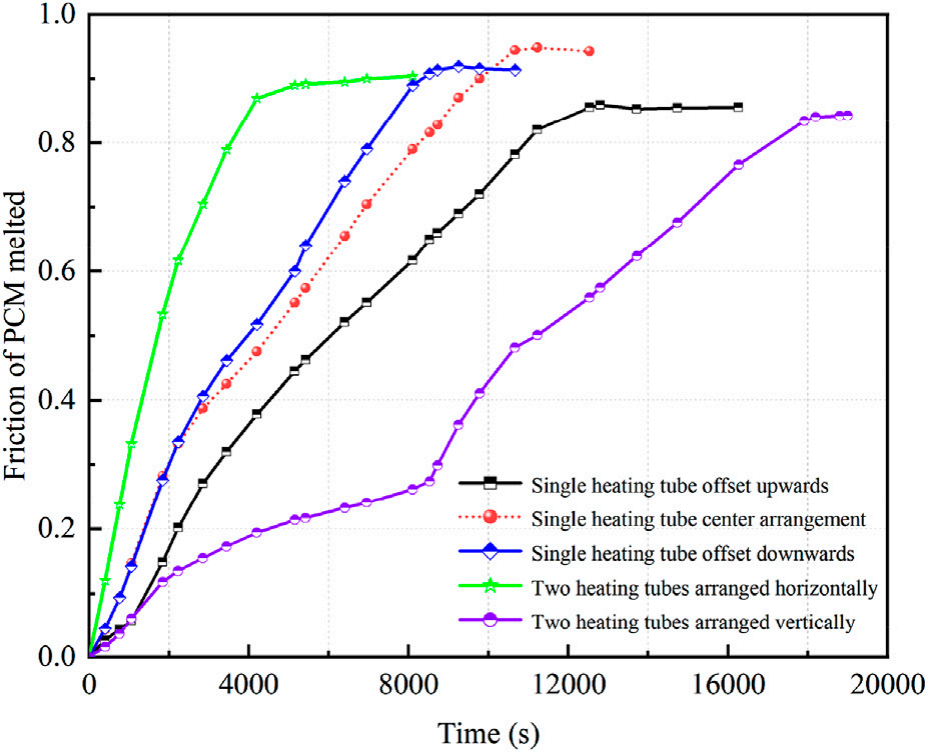
PCM volume fraction *versus* time curves for different heating tube arrangements.

Figure 13 depicts the temporal changes in the liquid PCM volume fraction across five distinct heating tube configurations, including one in a central reference position. In energy storage systems featuring a single heating tube, moving the tube 24 mm downward leads to optimal melting at 8,729 s. This shift marks a 20% enhancement in speed compared to the reference position. In contrast, shifting the tube 24 mm upward extends the melting duration to 12,536 s, indicating a 15% increase in melting time. Placing the heating tube lower augments the PCM volume affected by natural convection, extending the phase when convection enhances heat transfer. This results in a faster melting rate and a shorter overall melting duration. Conversely, raising the tube reduces the area influenced by convection, slowing the melting process. Dual heating tubes positioned horizontally complete PCM melting in 6,956 s, a notable 62% faster than the vertically oriented tubes requiring 18,200 s. The horizontal configuration promotes more efficient PCM melting due to improved heat distribution and convection flow. However, even with two tubes, the vertical alignment struggles with uneven heating and possible tube interference, causing it to lag behind some single-tube designs.

### 3.4 Experimental results

**FIGURE 14:**
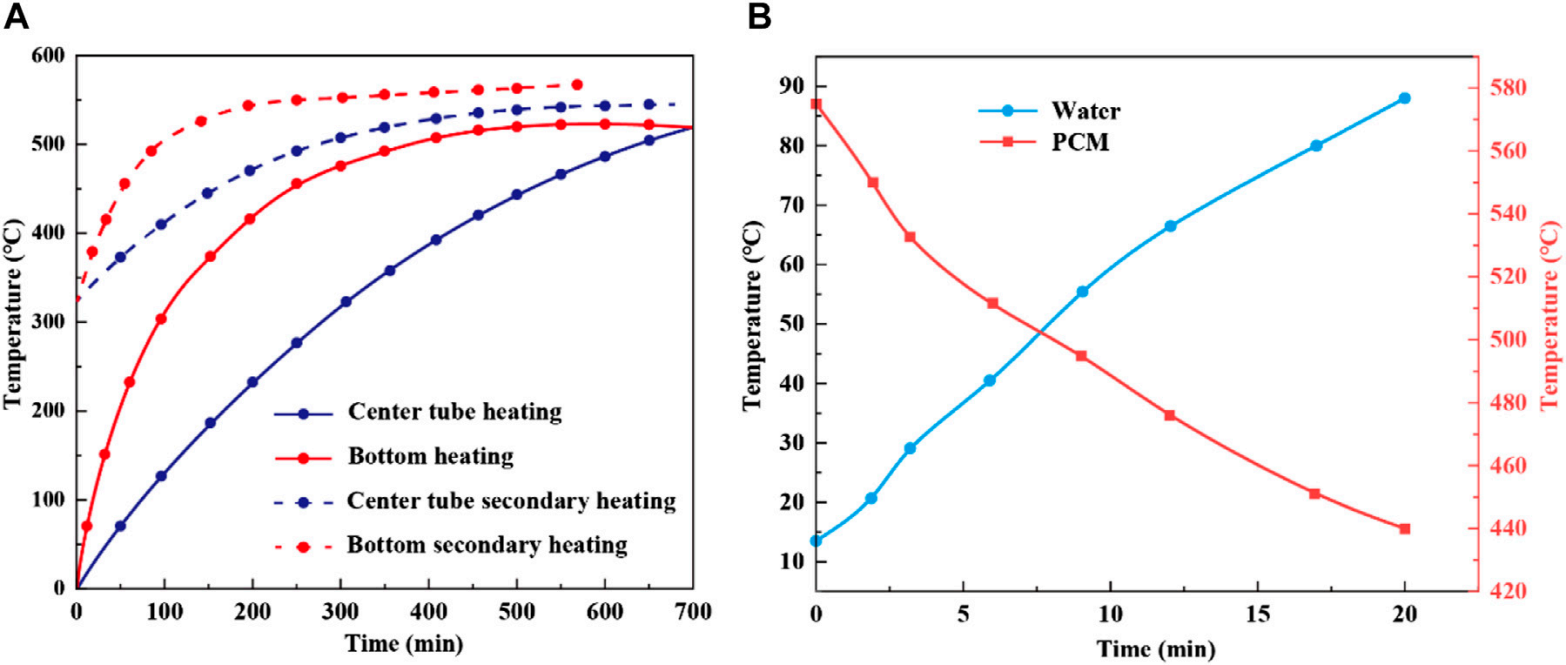
Temperature variations of PCM during thermal storage for two heating methods **(A)** and temperature comparison of PCM and water during heat release **(B)**.

In evaluating the temperature variations of PCM through both bottom heating and a horizontal dual-pipe setup, Figure 14A shows apparent differences in heating efficiency between these methods. Initially, the rate of temperature increase for the bottom heating method exceeds that of the dual-pipe setup, indicating a quicker temperature rise in the PCM. Specifically, bottom heating takes 10 h to fully charge the thermal storage device, achieving a peak temperature of 523°C for the PCM. In contrast, the dual-pipe method cannot reach this temperature within the same timeframe, highlighting its inefficiency. A second charging occurs after some PCM usage and a decrease to 323°C. The temperature rise with bottom heating is quicker than with the dual-pipe method, as depicted by the dashed line, emphasizing its enhanced efficiency during the recharging phase. Notably, after the second charge using the bottom heating method, the PCM temperature peaks at 563°C, a level the dual-pipe method does not achieve in the same period. Overall, the bottom heating method initially demonstrates a quicker temperature rise and retains its efficiency during the second charge. This is likely due to the direct heating of the PCM by the bottom method, which promotes bottom-to-top thermal convection, ensuring even and rapid heating of the entire PCM. Figure 14B displays the PCM and water temperature profiles during the endothermic phase when the thermal storage system heats a water vessel. Within 20 min, the temperature of 4.5 L of water rose from an ambient 20°C to approximately 88°C. Simultaneously, the temperature of the PCM decreased from an initial 575°C–440°C. Energy analysis indicates that about 66.3% of the PCM’s energy was utilized to heat the water. However, when subjected to unregulated external conditions, the thermal storage system encountered increased thermal losses, slightly reducing operational efficiency.

## 4 Conclusion

In thermal storage using PCMs, the method of heat application plays a pivotal role in determining efficiency. This study explored the advantages and limitations of bottom heating compared to central tube heating, emphasizing their critical roles in PCM optimization. The results indicate that bottom heating exhibits a progressive enhancement in natural convection as the liquid PCM region expands. By the time t = 6,100 s, this method results in the complete melting of the PCM, with the phase boundary becoming horizontal, denoting the end of the latent heat storage phase. This observed trend highlights the pronounced efficiency of bottom heating, particularly during the charging and recharging phases. Experimental data comes from the fact that bottom heating can achieve a peak temperature of 523°C in the PCM in 10 h. In contrast, central tube heating initiates PCM melting proximal to the tube due to direct heat conduction. Although central tube heating eventually leads to the total melting of the PCM, demonstrating its effectiveness, it does not match the speed of the bottom heating approach. Among the configurations tested, horizontally aligned dual heating tubes showcased optimal heat transfer. In practical applications using bottom heating, a 4.5 L water container experienced a swift temperature elevation from ambient to approximately 88°C within just 20 min. In summary, while central tube heating, especially with horizontally aligned dual tubes, has its merits, bottom heating demonstrates superior efficacy in the studied contexts. This research provides invaluable direction for advancing thermal storage systems that deploy PCMs, marking a significant step towards enhanced energy storage.

## Data Availability

The raw data supporting the conclusion of this article will be made available by the authors, without undue reservation.
